# The Effect of Acupuncture on Visual Function in Patients with Congenital and Acquired Nystagmus

**DOI:** 10.3390/medicines4020033

**Published:** 2017-05-23

**Authors:** Tilo Blechschmidt, Maike Krumsiek, Margarita G. Todorova

**Affiliations:** Department of Ophthalmology, University of Basel, Mittlere Strasse 91, CH-4031 Basel, Switzerland; tilo.blechschmidt@usb.ch (T.B.); maike.krumsiek@usb.ch (M.K.)

**Keywords:** acupuncture, nystagmus, contrast vison, visual function, nystagmography

## Abstract

**Background:** The aim of this study is to examine the short-term effect of visual function following acupuncture treatment in patients with congenital idiopathic nystagmus and acquired nystagmus (CIN and AN). **Methods:** An observational pilot study on six patients with confirmed diagnosis of nystagmus (three CIN and three AN patients (2♀, 4♂; mean age 42.67; SD ± 20.57 y)), was performed. Acupuncture treatment was done following a standardized protocol applying needle-acupuncture on the body and the ears. The treatment was scheduled with 10 sessions of 30 min duration over five weeks. To assess the effect of the treatment, we performed before, between, and after acupuncture objective measurement of the BCVA (EDTRS charts), contrast vision (CSV-1000, Vector Vision), nystagmography (Compact Integrated Pupillograph), complemented by evaluation questionnaires. A placebo non-acupuncture control group (Nr: 11, 22 eyes; 8♀, 3♂; mean age: 33.34 y (SD ± 7.33 y)) was taken for comparison. **Results:** The results showed that, following acupuncture treatment, CIN and AN patients showed improvement (SD± mean) in their binocular BCVA (baseline: 0.45 ± 0.36; between: 0.53 ± 0.34 and post-treatment: 0.51 ± 0.28), and in their monocular contrast sensitivity (baseline: 11.29 ± 12.35; between: 11.43 ± 11.45 and post-treatment: 14.0 ± 12.22). The post-/baseline-difference showed a significant improvement in contrast vision and in BCVA for CIN and AN patients, but not for controls (*p* = 0.029 and *p* = 0.007, respectively). The effect of the eye showed also, within CIN and AN, significant values for the examined parameters in the post-/baseline difference (*p* = 0.004 and *p* ≤ 0.001). Evaluated only binocularly, the respective between-/baseline and post-/baseline difference in the CIN and AN group showed significant values (*p* < 0.045). Two AN patients reported reduction of oscillations. Among general subjective symptoms, our patients reported reduction of tiredness and headache attacks, improvement of vision, and shorter sleep onset time. **Conclusion:** The applied acupuncture protocol showed improvement in the visual function of nystagmus patients and thus, in their quality of life. Further studies are mandatory to differentiate which group of nystagmus patients would benefit more from acupuncture.

## 1. Introduction

Nystagmus refers to a heterogeneous group of diseases, characterized by an involuntary rhythmic oscillation of the eyes. The resulting excessive movement of images on the fovea leads to a reduction of central vision. The onset time and the waveform characteristics of the nystagmus, categorize it into: congenital idiopathic nystagmus (CIN), which usually appears at birth or in early infancy; and acquired nystagmus (AN), presenting later in life. Contrary to the variety of distinctive waveforms described in recordings of the eye movements of CIN patients, the nystagmus waveforms are simpler in patients with AN. CIN can be idiopathic or associated with visual sensory abnormalities, as for instance, with congenital cataracts, retinal or optic nerve dystrophy/ hypoplasia, or it can be part of neurological disease or syndrome [1,2]. In order to exclude any underlying ocular or systemic pathology, in children with nystagmus, further electrophysiological laboratory tests—together with neurological work-up and neuro-radiological imaging—may be necessary. Neurological disease should be ruled out when the nystagmus is asymmetric in direction, unilateral by nature, or when its characteristics have changed over time.

Generally, treatment options for nystagmus include a refractive error correction and pharmacological and surgical interventions. Spectacles and contact lenses for correction of the underlying refractive error are the first choice to reduce optical aberrations and enlarge the retinal image and visual field, thus improving the quality of the retinal image [3,4,5]. Pharmacological agents used for nystagmus mitigation—such as GABA and glycine agonists—have proven to be effective in patients with CIN [6]. However, in cases of obviously abnormal head posture, eye muscle surgery remains an important option in order to shift the null-zone of the nystagmus into primary gaze, and thus to alleviate the neck/ocular torticollis [7,8,9].

Acupuncture treatment is still considered a non-mainstream therapeutic approach. Yet, its positive effect has been shown in a variety of degenerative and psychosomatic diseases, following cerebral and peripheral ischemia, and it was also effectively used to improve blood flow. For instance, in a mice model of Parkinson’s disease, the application of electro-acupuncture has proven to be effective in slowing the degeneration of dopaminergic neurons in the ventral midbrain [10]. Acupuncture has been also applied in various diseases involving psychosomatic status: such as anxiety, depression, and sleep disturbances [11]. There are also previous reports on the application of acupuncture in patients with nystagmus: vibratory and electrical stimulation of the face and neck were found to improve foveal fixation and thus, visual acuity in patients with CIN [12]. Blekher et al. reported a reduction of frequency and a decrease of slow phase velocities, as well as an improvement of foveolar fixation, following acupuncture in patients with CIN [13].

Particularly significant is the fact that patients with nystagmus often present unstable cerebral and vertebral blood flow [14]. As traditional acupuncture stimulation has shown its positive effect on systemic blood flow [15,16], it seems to be a promising supporting treatment in patients with nystagmus, as well.

The quality of life of patients with nystagmus is greatly dependent on the fluctuations of their nystagmus speed and velocity, and thus on the central fixation. Any attempt to stabilize their fixation, therefore, seems to be of benefit for them.

With this background in mind, we aimed at examining the effect of needle acupuncture on the body and the ears of nystagmus patients, following standardized acupuncture protocol. In addition, we evaluated which specific nystagmus patients the acupuncture drew greater benefit from the treatment.

## 2. Materials and Methods

This was an observational pilot study, in which all subjects received acupuncture, in order to determine a preliminary short-term efficacy or proof of principle of a standardized acupuncture protocol. The patients were recruited through the Department of Ophthalmology at the University of Basel (T.M.G.; B.T.).

The acupuncture protocol was developed for a group of nystagmus diseases, based on the extensive clinical experience of an ophthalmologist and a licensed, qualified, and well-trained acupuncturist (B.T.). A placebo non-acupuncture control group (Nr: 11, 22 eyes; 8♀, 3♂; mean age: 33.34 y (SD ± 7.33 y)) was taken for comparison, as the treatment is still controversial.

All procedures took place in the acupuncture unit at the Department of Ophthalmology at the University of Basel between July 2014 and May 2015. The study and data accumulation were in conformity with institutional requirements, and in accordance with the statements and principles of the declaration of Helsinki, as well as all governmental regulations.

### 2.1. Subjects

Patients suffering from congenital idiopathic nystagmus and acquired nystagmus (CIN and AN), followed at the Department of Ophthalmology (University of Basel, Basel, Switzerland), were enrolled in the study.

According to the clinical, diagnostic, and orthoptic findings, the patients were divided into the following groups:

- Patients with clinical characteristics of congenital idiopathic nystagmus (CIN), *N* = 3 (6 eyes),
- Patients with phenotypic characteristics of acquired nystagmus (AN), *N* = 3 (6 eyes).

Inclusion criteria for all patients were: congenital idiopathic nystagmus and acquired nystagmus; Caucasian origin.

Exclusion criteria were: above inclusion criteria not fulfilled; presence of ocular and/or systemic pathology other than nystagmus; currently using antidepressants, alcohol, or drugs; unwillingness to participate in the study, hyper-reactivity to acupuncture treatment.

Alarming signs and symptoms excluding any participation in the study and indicating instead further neurological, neurosensory, and imaging work-up were: history of onset after the age of four months, dissociated (asymmetric) form of nystagmus, preserved optokinetic nystagmus, presence of afferent pupillary defect, papilledema, and neurological symptoms like vertigo and nausea.

All nystagmus patients fulfilling our inclusion criteria underwent detailed ophthalmic examination including: refraction, best-corrected visual acuity at 2.5 m distance (BCVA), contrast vision at 2.5 m distance, nystagmography (Compact Integrated Pupillograph), intraocular pressure (Goldmann applanation tonometer), slit-lamp examination, biomicroscopy, and fundoscopy.

For each patient, BCVA was measured at distance with a standard decimal visual acuity Snellen charts. Contrast vision was evaluated at distance using a CSV-1000 colour vision test (CSV-1000, Vector Vision). The nystagmography was performed using compact integrated pupillograph (CIP, AMTech) and performed by the same experienced orthoptist (K.M.). Since the visual function in nystagmus patients could change from day to day or even during the day—and is thus linked to the nystagmus frequency, amplitude, and foveation periods during nystagmus—all examinations were performed in the morning. Also, in order to have reproducible measurements completed, the nystagmus was measured monocularly, in primary gaze, keeping fixation for distance. In this way, any additional effect of convergence on nystagmus characteristics could be excluded, as well. The patient was asked to keep both eyes during the examination open. Nystagmus latency was defined as delay between the onset of target movement and the initiation of target movement and nystagmus velocity was defined as the time taken to complete the saccade, once it was initiated.

Subjectively, the effect of acupuncture was evaluated using evaluation questionnaires in regard to changes in visual acuity, contrast sensitivity, as well as the presence of oscillopsias and their change following acupuncture.

For a fair comparison, our non-acupuncture control group also underwent measurements of: refraction, BCVA at 2.5 m distance, contrast vision at 2.5 m distance, intraocular pressure (Goldmann applanation tonometer), slit-lamp examination, biomicroscopy, and fundoscopy.

The acupuncture method followed a modified standardized protocol [17]: The acupuncture protocol consists of 10 sessions of 30 min duration, administered twice a week over a period of five weeks.

The scheduling of each patient and the complete orthoptic examinations were performed by the same experienced orthoptist (K.M.). Before the initial treatment appointment, the acupuncturist gave the patient a brief introduction outlining the duration and the course of treatment, as well as the possible complications. Each scheduled treatment session was initiated only after a short conversation with the patient and his answers to questions concerning his condition after the previous treatment and his present general condition.

### 2.2. Needle Acupuncture of the Body and the Ears

**Figure 1:**
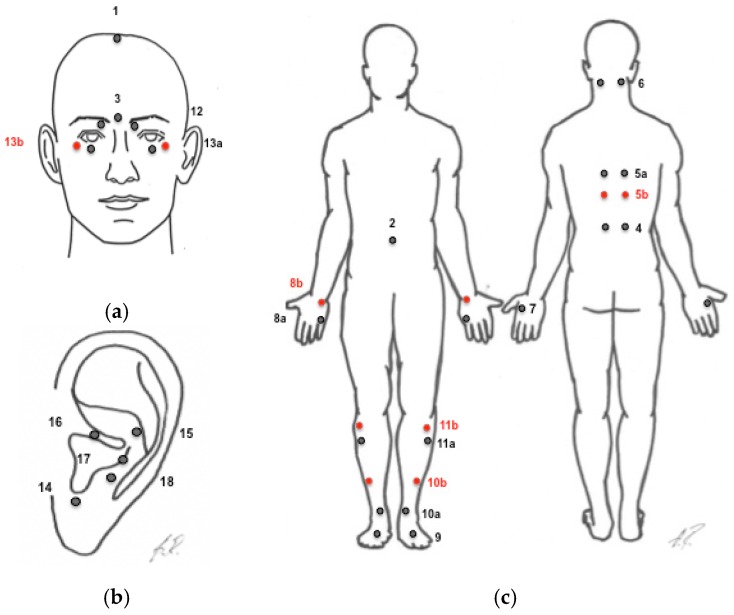
Needle acupuncture of the body and the ears was performed following standardized protocol (Table 1). The approximate location of the needles on the face and the ears are presented on (**a**,**b**); and on the body - on (**c**). The needles at LI-4 (He Gu), CV-6 (Qi Hai), LV-3 (Tai Chong), and all ear needles were manually stimulated once in each session after 15 min (+/−5 min). Treatment 1 (labelled in black) alternated with treatment 2 (labelled in red, see also in Table 1).

Sterile and disposable single-use needles of different sizes were used, namely Seirin B type needle No.3 (0.20) × 15 mm, No.5 (0.25) × 40 mm, No.8 (0.30) × 30 mm, Seirin Pyonex Press Needles P type 0.22 × 1,6 (Seirin Corporation, Shizuoka, Japan); Dong Bang needle DB106 (0.20) × 15, DB105G (0.20) × 25, Dong Bang Press Needles 0.20 × 2 × 1.0 (Dong Bang Acupuncture, Inc., Chungnam, Korea). The established protocol indicates the specific pre-selected points for all participants, needling depths, and manipulation techniques. The needles were applied by the same certified and experienced acupuncturist (BT). The standard points for all subjects were located around the eyes, on the head, ears, back, abdomen, arms, hands, lower legs, and toes and include: GV-20 (Bai Hui), ExHN-3 (Yin Tang), CV-6 (Qi Hai), UB-18 (Gan Shu), UB-20 (Pi Shu), UB-23 (Shen Shu), GB-20 (Feng Chi), LI-4 (He Gu), HT-7 (Shen Men), SI-3 (Hou Xi), LV-3 (Tai Chong), GB-34 (Yang Ling Quan), GB-37 (Guang Ming), SP-6 (San Yin Jiao), ST-36 (Zu San Li); local points: UB-1 (Jing Ming), ST-1 (Cheng Qi), ExHN-7 (Qiu Hou); ear points: Eye Point (24a), Liver Zone (97), Zero Point, Brainstem, and Point de Jerome (29b). Additionally, semi-permanent-needles were localized at following ear points: Liver Zone (29), Zero Point, and Brainstem. The needles were applied according to a standardized protocol (Figure 1, Table 1). Individual choice of acupuncture points was not allowed in contrary to the common Chinese Medicine (CM). Likewise, due to standardization, the number of applied needles exceeded the common practices of CM. The needle application sites were determined with respect to the CM standards. The aim was to produce an irradiating needle sensation (‘de qi’), if possible. The needles at LI-4 (He Gu), CV-6 (Qi Hai), LV-3 (Tai Chong), and all ear needles were manually stimulated once in each session after 15 min (+/−5 min). Additional CM therapeutic techniques—like electro-stimulation, heat lamps, music during treatment, etc.—were not applied.

### 2.3. Statistical Analysis

A statistical package IBM SPSS Statistics 22 was applied. To evaluate the effect of acupuncture on visual functions, a univariate ANOVA test was performed. In our statistical model, the group was taken as a fixed factor. Each pair of the tested objective methods (best-corrected visual acuity (BCVA, Snellen charts)), contrast vision (CSV-1000, Vector Vision), amplitude, and frequency of nystagmus (Nystagmography, Compact Integrated Pupillograph CIP, AMTech), was treated as a dependent variable.

The acupuncture effect on patients was analyzed at baseline (1: baseline), after five acupuncture sessions (2: between treatment), and at the end of the acupuncture protocol (3: post-treatment). Our results are presented as the mean difference for each of the tested parameters, with the respective standard deviation.

In addition, for each nystagmus patients and for each control subject, the difference between-/pre-treatment was calculated by extracting the between treatment value from the pre-treatment one (2-1). Correspondingly, the post-/baseline-treatment difference was calculated extracting the post-treatment value from the baseline-treatment one (3-1). The effect of acupuncture was assessed comparing the between-/baseline-treatment difference against the post-/baseline-treatment difference within nystagmus patients. P-values of less than 0.05 are considered statistically significant.

## 3. Results

An observational pilot study on six patients with clinical picture and confirmed diagnosis of nystagmus (three CIN and three AN patients (2♀, 4♂; mean age 42.67; SD ± 20.57 y)), was performed. A placebo non-acupuncture control group (Nr: 11, 22 eyes; 8♀, 3♂; mean age: 33.34 y (SD ± 7.33 y)) was taken, for comparison. The clinical characteristics of the patients are given in Table 2.

A subjective improvement of visual function, following the acupuncture treatment, was stated by all nystagmus patients. Evaluated in subgroups, two AN patients and one CIN patient reported reduction of oscillations (Table 2). Among the subjective general symptoms, our patients reported on reduction of tiredness and headache attacks, improvement in vision, and shorter sleep onset time.

**Figure 2:**
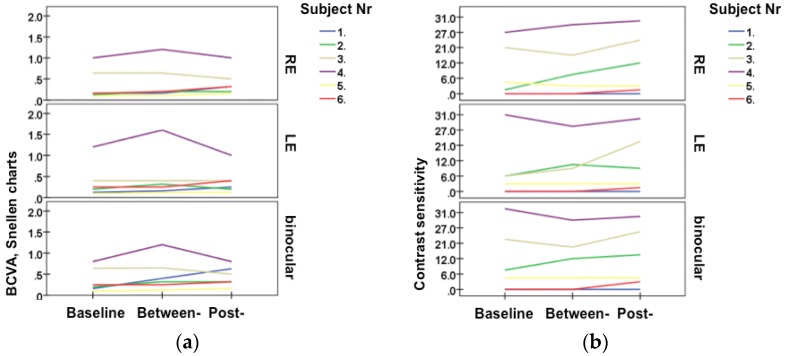
Line diagrams representing the individual patient’s examination data (presented separately for the RE: right eye; for the LE: left eye and for both eyes: binocular); (**a**) BCVA, Snellen charts; (**b**) Contrast sensitivity. The acupuncture effect was analyzed at the beginning (1: baseline-), after five acupuncture sessions (2: between) and at the end of the acupuncture treatment (3: post-).

Objectively, CIN and AN patients showed improvement (SD± mean) in their binocular BCVA (baseline: 0.45; ±0.36; between: 0.53; ±0.34 and post-treatment: 0.51; ± 0.28), in monocular contrast sensitivity (baseline: 11.29 ± 12.35; between: 11.43 ± 11.45 and post- treatment: 14.0 ±12.22; Figure 2a). Evaluating the between-/baseline-treatment difference against the post-/baseline-treatment difference, we found within CIN and AN patients a statistically significant increase in visual acuity (*p* = 0.002) and a statistically significant trend in improvement in contrast vision (*p* = 0.099; Figure 2b).

Compared to controls, CIN and AN patients showed a significant improvement in their contrast sensitivity and in their BCVA for the post-/baseline-treatment difference (*p* = 0.029 and *p* = 0.007, respectively). The effect of the eye also showed significant values between both groups for the examined parameters in the post-/baseline-treatment difference (*p* = 0.004 and *p* ≤ 0.001). These data pointed towards a different relation, when both the eye and the group effects were taken into account. Thus, we proceeded with evaluation of the examined parameters between groups in relation to the examined eye. Within CIN and AN patients, now we could document improvement in their binocular BCVA and contrast sensitivity for both, the between-/baseline-treatment difference, as well as for the post-/baseline-treatment difference with respective values, as follows: 0.044, 0.029; 0.061; 0.006. Here, the corresponding monocular values did not reach statistically significant values (*p* > 0.005).

All six CIN and AN patients demonstrated reduction of the mean amplitude and frequency of the nystagmus, following acupuncture (Table 3). No change of the direction of the nystagmus was recorded in any patient.

**Figure 3:**
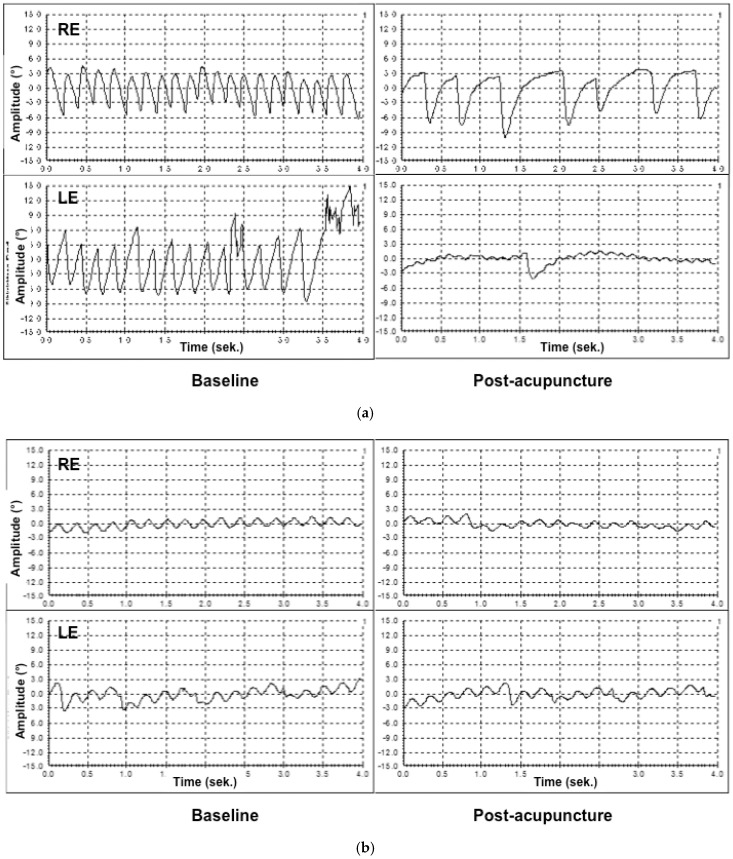
Nystagmography pictures in two patients (**a**); (**b**) baseline and following acupuncture treatment, as described bellow.

As an example, in the case with CIN, hyperopia and macula hypoplasia (patient 2, Table 2) the waveform characteristics changed from horizontal conjugate nystagmus with pendular velocity to jerk disconjugate nystagmus, with descending velocity slow phase more pronounced on the right eye (Figure 3a). Here, even if no oscillations were reported, the reduction of the nystagmus amplitude and frequency explained the subjective and objective improvement of monocular and binocular visual acuity of the patient, as well as in the contrast vision. As it is shown in Figure 3a, the frequency and the amplitude of nystagmus (No. saccades/amplitude/velocity) improved much following acupuncture treatment: from 19/6.5°/3.5 s, right and 15/9°/3.5 s, left to 6/6°/3.5 s, right and 2/7°/3.5 s, left. On the contrary, in the case with pendular nystagmus and multiple sclerosis (patient 6, Figure 3b), no improvement in the velocity of nystagmus was measured, but in the amplitude, on the left. Here, by 15 objectively stable saccades at baseline and at post-treatment, a slight reduction in the amplitudes from 4.5° to 4° on the right eye and from 5.5° to 5° on the left eye was recorded (Figure 3b). In addition, the patient reported subjectively on reduction of the oscillations, as well as on improvement in her binocular and monocular vision and contrast sensitivity (Table 2).

Figure 3a shows an example of nystagmography pictures of a 23-year-old male patient with congenital idiopathic nystagmus, hyperopia, and macula hypoplasia. The patient reported no oscillations. The nystagmography showed, however, a significant reduction of the nystagmus amplitude and the velocity of nystagmus (No. saccades/amplitude/velocity) improved significantly following acupuncture treatment: from 19/6.5°/3.5 s, right and 15/9°/3.5 s, left to 6/6°/3.5 s, right and 2/7°/3.5 s, left. The findings confirm his subjective and objective improvement of monocular and binocular visual acuity, and contrast sensitivity.

Figure 3b shows an example of nystagmography pictures of a 26-year-old female patient with idiopathic pendular nystagmus as part of multiple sclerosis. Even if no improvement in the velocity of nystagmus (15 saccades at baseline and at post-treatment) was measured, an amplitude reduction from 4.5° to 4 on the right eye and from 5.5° to 5° on the left eye could be found. This finding explains the subjectively reported oscillations’ reduction, as well as vision- and contrast sensitivity improvement (Table 2).

Neither ocular, nor systemic adverse effects, were reported by any nystagmus patient. All patients included in the study indicated a willingness to repeat the acupuncture treatment.

## 4. Discussion

Results of our pilot study on patients suffering from nystagmus, confirmed subjective and objective improvement in their visual function, following standardized acupuncture protocol. Our data also demonstrated a dose-response relation: post-acupuncture, the effect of on visual acuity and contrast vision is more pronounced than the effect between-acupuncture, always using the baseline data for comparison.

This is not an unexpected finding, since previous reports on nystagmus patients treated with acupuncture also measured improvement of their foveal visual acuity and contrast sensitivity [12,13]. However, the studies reported until now have been performed on patients with CIN [12,13]. Here, the study of Blekher et al., examined the effects of acupuncture of the neck and face, reporting a reduction of frequency and a decrease of slow phase velocities, as well as an improvement of foveolar fixation, following acupuncture in patients with CIN. The improvement of these foveation periods has been thought to enhance visual acuity. Thus, the pathophysiologic mechanism of the improvement of the underlying CIN was supposed to be produced through the afferent stimulation of the reticular formation and vestibular nucleus via acupuncture of the neck and the face [12].

A novel finding in the present study was the fact that AN patients seemed to benefit from the acupuncture more. A possible explanation for this finding might be the labile nature of the CIN: the nystagmus may be exacerbated with visual effort or when fixating an imaginary target, and on the contrary may reduce with inattention or fatigue. It is noteworthy that the placement of the eyes in convergence gaze angle frequently dampens the nystagmus, which in fact is used as a treatment modality applying prism correction or divergence surgery. The latter explains why the binocular effect on visual function over time was more pronounced in our study and stays in agreement with previous studies. Nevertheless, to exclude the influence of the gaze direction and near fixation on nystagmography, and following the manufacturer’s protocol (CSV-1000, Vector Vision; CIP, AMTech), we examined all six patients with the gaze directed for distance, keeping both eyes opened, while performing nystagmography monocularly.

We also found that even if the direction of the nystagmus showed no difference in either of nystagmus subgroups, patients with AN showed reduction in the nystagmus amplitude and frequency, and thus in oscillations, following acupuncture treatment. As a consequence, their central vision and contrast sensitivity seems to have improved more. On the contrary, patients with CIN showed almost stable amplitude and frequency of their nystagmus pattern.

Since patients with nystagmus often present unstable blood flow [14,16], the effect of acupuncture on systemic and ocular blood flow seems to explain in part the present finding. In agreement, studies on electro-acupuncture in rabbits with vertebrobasilar insufficiency showed improvement in their vestibulo-ocular reflex, through improvement of the basilar artery hemodynamic, inner ear blood flow, and blood viscosity [15]. Moreover, the traditional acupuncture stimulation has shown its positive effect on systemic blood flow, an effect, which supposedly is in part mediated by the central nervous system [16].

Even though in different ways, both nystagmus subgroups benefited from the acupuncture treatment. Acupuncture induced more foveation in CIN while mainly mitigating ocular-blood flow disturbances in AN patients. In this regard, our conclusions remain to be elucidated and elaborated upon in further studies.

## 5. Conclusions

Briefly, the applied acupuncture protocol showed improvement of the visual function and systemic condition of our nystagmus patients and was well tolerated. However, the long-term effect of this complementary therapy needs to be evaluated in further studies.

## Figures and Tables

**Table 1:** All acupuncture points, the alternation in treatment (labeled in red, see also in Figure 1) and the stimulation duration. Standardized needles acupuncture protocol was applied as shown in Figure 1.

Acupuncture Study Protocol			Laterality
Treatment Modality	Treatment 1 (a)	Treatment 2 (b)	
Needle Nr:	Alternating with Treatment 2	Alternating with Treatment 1	
1	GV20 (Bai Hui)		Median
2	CV6 (Qi Hai)		Median
3	Ex-HN 3 (Yin Tang)		Median
4	UB23 (Shen Shu)		BL
5 (a/b)	UB18 (Gan Shu)	UB20 (Pi Shu)	BL
6	GB20 (Feng Chi)		BL
7	LI4 (He Gu)		BL
8 (a/b)	SI3 (Hou Xi)	HT7 (Shen Men)	BL
9	LV3 (Tai Chong)		BL
10 (a/b)	SP6 (San Yin Jiao)	GB 37 (Guang Ming)	BL
11 (a/b)	GB 34 (Yang Ling Quan)	ST36 (Zu San Li)	BL
12	UB1 (Jing Ming)		BL
13 (a/b)	ST1 (Cheng Qi)	EX-HN7 (Qiu Hou)	BL
**Additional Ear Acupuncture Points** **(Alternately, Starting with the Right Ear)**			
14	Eye Point (24a)		UL
15	Liver Zone (97)		UL
16	Zero Point		UL
17	Brainstem		UL
18	Point de Jerome (29 b)		UL
**+ One Semi-Permanent Needle (Press Tack Needle)** **(Alternately, Starting with the Left Ear, Points in the Order Specified Bellow):**			
15	Liver Zone (97)		UL
16	Zero Point		UL
17	Brainstem		UL
**Needle Stimulation (after 15 min) at the Following Points:**			
1	GV20 (Bai Hui)		s. above
2	CV6 (Qi Hai)		s. above
7	LI4 (He Gu)		s. above
9	LV3 (Tai Chong)		s. above
14–18	Ear Points		s. above
**Duration of Needle Stimulation:**			
30 min			

**Table 2:** Clinical characteristics of patients included in the study.

Patient		Nystagmus Characteristics, Ophthalmic/Systemic Diagnosis	Subjective Findings: Before versus after Acupuncture Treatment		
Nr:	Age (y), Gender		BCVA	Contrast Vision	Oscillations
Case 1	51, m	Consecutive exotropia and decompensated latent nystagmus after Botox injection (2014) and strabismus surgery, left eye (2015) for congenital esotropia, latent nystagmus, and dissociated vertical deviation.	Monocular and binocular improvement	Stable	Reduction
Case 2	23, m	Congenital idiopathic nystagmus. Hyperopia. Macular hypoplasia.	Binocular improvement	RE > LE improvement	No
Case 3	20, m	Horizontal pendular nystagmus, latent compound. Accommodative convergence excess esotropia and amblyopia, left eye. Optic disc hypoplasia. Status after surgery for neuroepithelial cyst, right lateral ventricle. Occipital para-ventricular: atrophy and gliosis	Worsening	Improvement	No
Case 4	70, f	Acquired torsional down-beat nystagmus after brain stem ischemia.	Stable	Stable	Reduction
Case 5	39, m	Congenital idiopathic nystagmus. Oculocutaneous Albinism.	Slight improvement	Stable	No
Case 6	26, f	Pendular nystagmus. Multiple sclerosis.	Slight improvement	Slight improvement	Reduction

**Table 3:** Summarized nystagmography results of all patients before- and after acupuncture treatment. In order to reduce the influence of the head position and the effect of convergence, each patient was asked to keep both eyes during the examination open while the measurement was performed monocularly, in primary gaze, keeping fixation for distance.

Patient Nr/Age/Gender	Eye	Saccades within 4 Sec. (before-/after Acupuncture)	Amplitude, ° (before-/after Acupuncture)	Velocity, Sec. (before-/after Acupuncture)	Subjective Evaluation after a “Washout” Time Following Acupuncture
1/51/m	RE	16/11	9/8	4/4	Stable since acupuncture
	LE	9/10	4/4.5	4/4	
2/23/m	RE	36/18	6.5/3.5	4/4	No control examination has been done
	LE	24/25	6/7	4/4	
3/20/m	RE	20/7	9/7	4/4	Still stable three months after acupuncture
	LE	17/27	18/11	4/4	
4/70/f	RE	Not possible objectively to be analyzed, however reduced oscillations following acupuncture			Stable since acupuncture
	LE				
5/39/m	RE	Not possible to analyze/11	Not possible to analyze/6.5	4/4	No control examination has been done
	LE	Not possible to analyze/15	Not possible to analyze/17	4/4	
6/26/f	RE	17/17	4.5/4	4/4	Stable since acupuncture
	LE	17/17	5.5/5	4/4	
